# Prenatal Depressive Symptoms, Self-Rated Health, and Diabetes Self-Efficacy: A Moderated Mediation Analysis

**DOI:** 10.3390/ijerph192013603

**Published:** 2022-10-20

**Authors:** Sandraluz Lara-Cinisomo, Julio Ricardo Loret de Mola, Kendra Flores-Carter, Karen M. Tabb, Kristina Roloff

**Affiliations:** 1 Department of Kinesiology and Community Health, University of Illinois Urbana-Champaign, 1206 S. Fourth Street, Champaign, IL 61820, USA; 2 Department of Obstetrics and Gynecology, Southern Illinois University School of Medicine, 415 N. 9th St. Suite 6W100, Springfield, IL 62794, USA; 3 Department of Social Work, College of Behavioral and Social Sciences, California Baptist University, 8432 Magnolia Avenue, Riverside, CA 92504, USA; 4 School of Social Work, University of Illinois Urbana-Champaign, 1010 W. Nevada St., Urbana, IL 61801, USA; 5 Department of Women’s Health, Arrowhead Regional Medical Center, 400 N. Pepper Avenue, Colton, CA 92324, USA

**Keywords:** diabetes, self-efficacy, depressive symptoms, self-rated health, pregnant persons

## Abstract

Background: Diabetes leads to risk for pregnant persons and their fetuses and requires behavioral changes that can be compromised by poor mental health. Poor self-rated health (SRH), a reliable predictor of morbidity and mortality, has been associated with depressive symptoms and lower self-efficacy in patients with diabetes. However, it is unclear whether SRH mediates the association between depressive symptoms and self-efficacy in pregnant patients with diabetes and whether the healthcare site moderates the mediation. Thus, we sought to test these associations in a racially and ethnically diverse sample of pregnant individuals diagnosed with diabetes from two clinical settings. Materials and methods: This was an observational, cross-sectional study of 137 pregnant individuals diagnosed with diabetes at two clinical study sites. Participants self-administered a demographic questionnaire and measures designed to assess depressive symptoms, SRH in pregnancy, and diabetes self-efficacy. A moderated mediation model tested whether these indirect effects were moderated by the site. Results: The results show that SRH mediated the association between depressive symptoms and diabetes self-efficacy. The results also showed the site moderated the mediating effect of SRH on depressive symptoms and diabetes self-efficacy. Conclusions: Understanding the role of clinical care settings can help inform when and how SRH mediates that association between prenatal depressive symptoms and self-efficacy in diabetic patients.

## 1. Introduction

According to the Centers for Disease Control (2018), 1–2% of individuals who identify as women have type 1 or 2 diabetes, and approximately 6–9% of pregnant people will develop gestational diabetes. Between 2000 and 2010, gestational diabetes increased by 56%, and the percentage of women with type 1 and type 2 diabetes before pregnancy increased by 37% [1]. Type 2 diabetes is prevalent among minority ethnic groups, including people of African, Black Caribbean, South Asian, Middle Eastern, Central, and South American family origin [2,3]. Type 2 diabetes is projected to affect 693 million people worldwide by 2045 [4]. Diabetes increases the risks of adverse pregnancy outcomes for both parent and child, including preeclampsia, polyhydramnios, cesarean delivery, premature birth, neonatal hypoglycemia, birth defects, respiratory distress syndrome, and hyperbilirubinemia in the neonate [5,6,7,8]. Gestational diabetes also has been linked to long-term adverse health outcomes for pregnant people and their offspring [5,6,7,8]. Achieving adequate glycemic control is the cornerstone of preventing short- and long-term adverse health outcomes in people with diabetes during pregnancy. Treatment typically consists of lifestyle, behavioral and dietary changes, home glucose monitoring, and medical therapy with oral antihypoglycemics and/or insulin for persistent hyperglycemia. Though screening for diabetes in pregnancy is common, there is wide heterogeneity in reported improvements in pregnancy outcomes with treatment [5,6]. Adequate glycemic control during pregnancy has been demonstrated to reduce complications. Still, barriers to healthcare access, racial and ethnic disparities, including a higher prevalence of diabetes among people of color, and maternal comorbidities, such as mental health issues, may limit a person’s ability to achieve adequate blood glucose control [2,9,10]. Despite the known risks for poor outcomes, few studies have investigated factors simultaneously addressing diabetes and perinatal mental health.

### 1.1. Self-Rated Health

Self-rated health (SRH) considers an individual’s perception of their health and wellness. Self-rated health is a widely used measure of health that predicts morbidity, mortality, and health services [11,12]. Poor SRH is associated with a higher risk of type 2 diabetes [13,14]. Schytt and Hildingsson [15] found that SRH may decrease during pregnancy and postpartum or one year after giving birth. Others found an association between low SRH and perinatal depressive symptoms [16]. In a sample of Latina women, Lara-Cinisomo [17] found an association between diabetes, perinatal depression, and SRH, with worse SRH during pregnancy. The findings suggest that SRH can be a critical factor to explore in prenatal individuals with diabetes. Still, SRH’s association between depressive symptoms in pregnancy and diabetes self-efficacy is not fully explored.

### 1.2. Diabetes Self-Efficacy and Depression

Self-efficacy, defined as the level of self-confidence required to perform a specific behavior within their ability efficiently, is one of the most significant factors in behavior change to strengthen the proper management of diabetes [18,19]. Self-efficacy increases adherence to blood glucose monitoring, diet, insulin injections, and exercise [20]; and plays a pivotal role in successful diabetes management [21]. However, the presence of mental health disorders among individuals with diabetes may limit a person’s ability to perform diabetes self-care behaviors [22], including being physically active, monitoring glucose, controlling diet, and adhering to medications [7,23]. People with depression experience functional decline, limiting effective lifestyle changes vital for diabetes self-care management [24,25]. Depression during pregnancy is critical given the global prevalence [26,27,28] and risk for the birthing person and infant [29,30,31]. Therefore, evaluating the associations between psychological well-being and diabetes self-efficacy during pregnancy is critical.

### 1.3. Research Objectives

**Figure 1:**
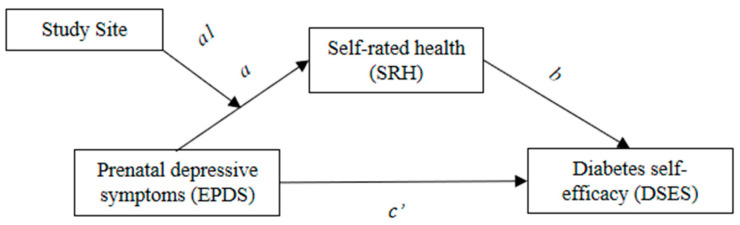
The figure shows the hypothesized mediating effect of self-rated health on the association between prenatal depressive symptoms and diabetes self-efficacy and the proposed moderating effect of the study site.

This study aimed to test the mediating effect of SRH on the association between depressive symptoms and diabetes self-efficacy in a sample of racially and ethnically diverse pregnant people. Because the healthcare setting might affect the mediating associations, we conducted a moderated mediation analysis. We hypothesized that SRH would mediate the association between depressive symptoms and self-efficacy. We also hypothesized that there would be moderated mediation, where mediation differed by study site (see Figure 1).

## 2. Materials and Methods

**Figure 2:**
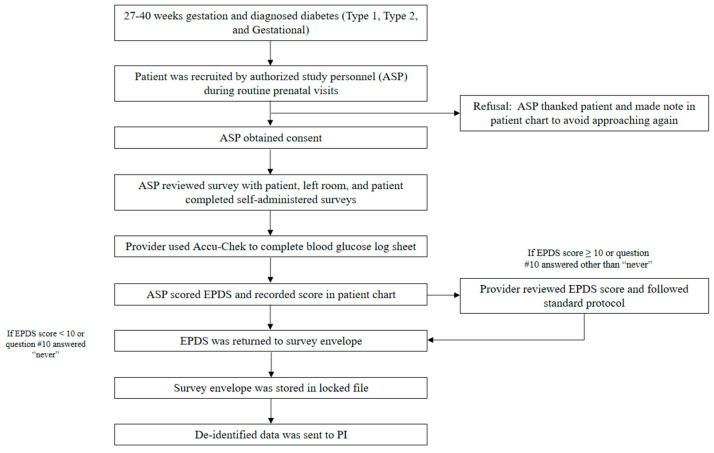
Participant recruitment flowchart.

This was an observational, cross-sectional study of 137 racially and ethnically diverse pregnant individuals diagnosed with Type 1, Type 2, or gestational diabetes mellitus (GDM) at two clinical study sites. Patients between 27 and 40 weeks of gestational age and diagnosed with diabetes were approached about the study by approved healthcare staff. Patients consented to participate, and if written consent was granted, they self-administered a short demographic questionnaire and the survey items described below (see Figure 2). Participants were not compensated. Data collection was conducted between November 2017 and March 2020. Data collection ended at the start of the pandemic.

The two study sites were located in Southern California (CA), and Central Illinois (IL). The California site is a teaching, safety net hospital with a predominantly Latina population. Over 90% of the patients are considered medically indigent or are enrolled in the state Medicaid program (MediCal), unpublished data. This site follows the California Diabetes and Program, Sweet Success Guidelines for Care [32]. Pregnant people with pre-existing diabetes (Type 1, 2, or gestational) or a new diagnosis of gestational diabetes were referred for education to a certified diabetes educator nurse, who recruited them for this study at an initial or return visit. Obstetricians, in consultation with on-site Maternal-Fetal Medicine (MFM) specialists, managed care of the pregnancy, comorbidities, and delivery. Screening for diabetes or GDM is performed with the International Association of Diabetes in Pregnancy Study Groups criteria with a first-trimester hemoglobin A1c and 24–28 week 2-h glucose challenge test [33,34]. In contrast, the IL site utilizes the Carpenter Coustan method, including a risk-based screening approach during the first trimester [35]. Patients are assessed at their first prenatal visit, and an additional 1-h oral glucose tolerance test (OGTT) is ordered if the patient is at risk for gestational diabetes mellitus (GDM)—including a history of GDM, a history of a macrocosmic infant in a prior pregnancy, a family history of diabetes mellitus, obesity, etc. Alternatively, a 1-h OGTT is administered at 24–28 weeks gestation. A referral to a certified diabetes care and education specialist nurse is given to all patients who fail this screening test. If diabetes is uncontrolled and medical management is required, the patient is referred to MFM.

To be eligible to participate, patients had to be 18–45 years of age, have a singleton pregnancy, be at least 27 weeks pregnant, be diagnosed with diabetes (Type 1, Type 2, or GDM), and be able to speak, read and write in English or Spanish. Individuals with end-stage renal disease, dementia, or blindness were excluded because these conditions could interfere with survey completion.

### 2.1. Measures

The following is a description of the self-administered measures. All measures were available in English and Spanish. Individuals completed the survey using their preferred language.

#### 2.1.1. Demographic Questionnaire

This instrument inquired about the patient’s age, marital status, education, annual family income, race and ethnicity, history of depression, age of diabetes diagnosis, and other health histories.

#### 2.1.2. Edinburgh Postnatal Depression Scale (EPDS)

This 10-item widely used instrument screens for post-childbirth depressive symptoms and has also been shown to be valid during the prenatal period [36]. Responses were based on a 4-point scale (0, 1, 2, and 3). After reverse scoring several items, responses were summed to produce a total score, with a maximum score of 30. Subjects with scores ≥ 10 were classified as at risk for depression [37,38]; a question specific to harming oneself is also included in the measure. If a patient indicated any choice other than ‘never,’ their provider was notified immediately, and the standard protocol for immediate treatment or referral was followed (see Figure 2). None of the participants reported anything other than ‘never’ having suicidal ideation. The EPDS has been used with diverse populations, including Spanish-speaking women [39]. The Cronbach’s alpha for the scale was α = 0.84.

#### 2.1.3. Diabetes Self-Efficacy Scale (DSES)

This 8-item scale includes questions regarding the extent to which respondents feel confident about their nutrition, exercise, glucose control, and disease management [40]. Participants are asked to rate their confidence on a 10-point Likert scale from 1 “Not at all confident” to 10 “Totally confident.” The score is the mean of items, with higher scores indicating higher confidence or self-efficacy. The scale is available in Spanish, the original language of the instrument, and English. Both measure versions are reliable and valid for assessing self-efficacy in diabetes management [41]. The Cronbach’s alpha for the scale was α = 0.88.

#### 2.1.4. Self-Rated Health (SRH)

SRH was measured through a single question, “Compared to other people your age, how would you describe the state of your physical health since you’ve been pregnant.” Responses were based on a 5-point Likert scale from Poor (1) to Excellent (5) and reversed coded for analytic purposes. This SRH measure is a subjective predictor of mortality similar to objective health [42]. This measure has also been used with diabetic populations, including pregnant people [17,43]

### 2.2. Statistical Analysis Plan

Descriptive statistics were computed for all study variables using SPSS 28.0. Categorical variables were summarized with frequencies and percentages. Means and standard deviations for continuous variables were computed. Fisher’s exact test, *t*-tests, and chi-square determined associations between dichotomous and categorical demographic characteristics and the outcome (DSES). Unadjusted linear regression tested associations between continuous demographic variables and DSES. Site-level differences in demographic characteristics, prenatal depression (PND measured using the EPDS scores), and diabetes self-efficacy score (DSES) were also explored using bivariate analyses. Mediation was tested in a model to assess the significant effect of SRH on the association between EPDS and DSES. EPDS was the predictor, SRH was the mediator, and DSES was the outcome variable. A moderated mediation model was used to examine whether the site moderated these indirect effects. The model included covariates significantly associated with the outcome (DSES). Mediation and moderated mediation were tested using Mplus using full information maximum likelihood [44]. All 137 subjects were used in the analyses. Unstandardized coefficients were used to estimate the mediation and moderated mediation. Inferences on indirect effects were tested using a bootstrap approach with 5000 samples [45]. Bias corrected bootstrap standard errors, and 95% confidence intervals of the direct and indirect effects were calculated. A 95% confident interval that does not include zero indicated that parameters were statistically significant.

## 3. Results

The sample demographic characteristics are reported in Table 1. The mean age was 30.47 (SD = 6.24), and the age of diabetes diagnosis was slightly younger, 28.60 (SD = 7.38), which was significantly different [*t* (107) = 40.283, *p* < 0.001]. Half the sample self-identified as Hispanic/Latina, a third were single, more than half worked at least part-time, and nearly half of the sample had more than high school education. Given the difference in the two study sites, we examined differences in demographic characteristics by site. There was a significant difference in the mean age of diagnosis by site [*t* (106) = 2.129, *p* = 0.036], with individuals at the IL site diagnosed at a younger age, on average. There was also a significant association between language and site (*p* < 0.001); Spanish data collection was unavailable at the IL site. There was a significant association between race/ethnicity and site [χ^2^ (4) = 94.031, *p* < 0.001]. The CA site had a higher proportion of Hispanic/Latina participants. In contrast, IL had a higher proportion of non-Hispanic White participants. Employment status and a history of depression diagnosis were also significantly associated with the site [χ^2^ (3) = 9.250, *p* = 0.026 and χ^2^ (3) = 25.113, *p* < 0.001], with a higher proportion of employment and a history of depression in IL. A higher proportion of individuals in IL versus CA met the cut-off for at-risk depression (18.2% versus 11%, respectively), a difference that was not statistically significant (*p* = 0.318).

### 3.1. Depressive Symptoms, Self-Rated Health, and Diabetes Self-Efficacy

As shown in Table 2, mean EPDS scores were low (5.18, SD = 4.37). The mean DSES score for the entire sample was 8.05 (SD = 1.70). However, the average SRH was considered low at 2.92 (SD = 1.03). Unadjusted regression analysis indicated that race/ethnicity, age, and site were significantly associated with the outcome (DSES). Individuals who identified as Latina or biracial reported significantly higher DSES compared to non-Hispanic White individuals (*B* = 1.18, *t* (130) = 3.74, *p* < 0.001 and *B* = 1.74, *t* (130) = 2.08, *p* = 0.040). There was also a significantly positive association between age and DSES (*B* = 0.06, *t* (133) = 2.54, *p* = 0.013). Lastly, there was a significant difference by site (*B* = −1.08, *t* (133) = −3.79, *p* < 0.001), with higher mean DSES scores among individuals in CA versus IL. However, chi-square tests showed that race and ethnicity were significantly associated with site. Therefore, race and ethnicity were not included in the models. Thus, age was the only covariate in the models.

We determined whether there were differences in the outcome (DSES) and the predictor (EPDS), and mediator variables (SRH) between the study sites. While individuals at the IL site had slightly higher mean EPDS scores, the difference was not statistically significant [*t* (129) = −1.935, *p* = 0.055]. Differences in mean SRH scores were not statistically different by study site [*t* (134) = 1.117, *p* = 0.266]. There was a significant difference in DSES by site, with individuals in the CA reporting significantly higher mean DSES [*t* (133) = 3.789, *p* < 0.001] compared to those in IL. EPDS scores were significantly and negatively correlated with SRH (*r* = −0.368, *p* < 0.001) and DSES (*r* = −0.393, *p* < 0.001). SRH was positively and significantly correlated with DSES (*r* = 0.311, *p* < 0.001).

### 3.2. Mediation Analysis

Findings from the mediation analysis revealed that SRH mediated the association between EPDS and DSES (see Table 3; Estimate = −0.037, SE = 0.016, 95% CI = −0.077, −0.011). The results also show a negative association between depressive symptoms (EPDS) and SRH (Estimate = −0.09, SE = 0.019, 95% CI = −0.129, −0.053). A negative association between EPDS and DSES was also observed (Estimate = −0.075, SE = 0.035, 95% CI = −0.148, −0.009). The findings also revealed a positive association between SRH and DSES (Estimate = 0.407, SE = 0.157, 95% CI = 0.099, 0.715).

### 3.3. Moderated Mediation Analysis

**Figure 3:**
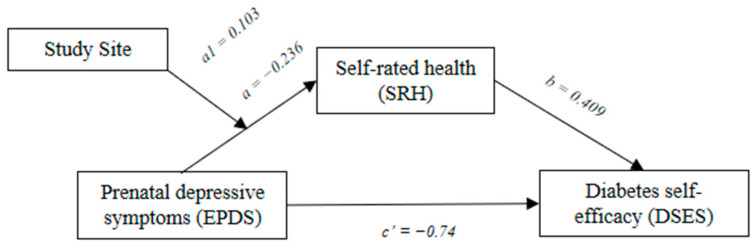
The figure shows the moderated mediation of site.

Regression analysis testing the joint effect of site and EPDS on SHR showed that the study site moderated the effect of EPDS on SHR (*F* = 8.33, *p* = 0.005). The results from the moderated mediation reported in Table 4 and Figure 3 show that the indirect effect of SRH on the association between EDPS and DSES differed between the two sites (Estimate = 0.103, SE = 0.039, 95%CI = 0.028, 0.182). The mediating effect of SRH was significant in the CA site (Estimate = −0.054, SE = 0.024, 95% CI = −0.11, −0.014), but not the IL site (Estimate = −0.012, SE = 0.013, 95% CI = −0.046, 0.009). The total effect was significant in CA (Estimate = −0.129, SE = 0.038, 95% CI = −0.211, −0.064) and IL (Estimate = −0.086, SE = 0.037, 95% CI = −0.162, −0.017).

## 4. Discussion

This study identified the mediating role of SRH in the association between depressive symptoms and diabetes self-efficacy among pregnant individuals with diabetes. While the empirical evidence shows that depressive symptoms are associated with lower SRH and lower diabetes self-efficacy, the role of SRH in these associations has not been established. Thus, this novel study tested our hypothesis that SRH would mediate the association between depressive symptoms and diabetes self-efficacy. In doing so, we also hypothesized that depressive symptoms would be associated with lower SRH and diabetes self-efficacy. Because the sample was drawn from two clinical settings, we tested the moderated mediation of site. The findings supported our assumptions.

The analyses revealed that SRH mediated the association between depressive symptoms and diabetes self-efficacy. However, we also found that site moderated the mediation. In other words, the mediating effect of SRH differs by clinical setting. It is critical to note that race and ethnicity were correlated with the study setting; most Hispanic/Latinas were located in CA, and most non-Hispanic Whites were in IL. Therefore, we cannot determine whether the sample population, the geographic area, the clinical approach, or the characteristics of the clinical site explain the moderating effect of study site. Still, this cross-sectional study of a diverse sample of pregnant people diagnosed with diabetes indicated that depressive symptoms were significantly and negatively associated with diabetes self-efficacy, even after controlling for age, which was the only demographic variable associated with the outcome variable. Still, while these findings support previous studies that showed similar associations [46], we must acknowledge that the association was observed in one of the two clinical settings, suggesting that characteristicsof the setting or the clinical population matter. As noted previously, patient race and ethnicity were significantly correlated with study site. As Table 1 shows, most Hispanic/Latina patients were located in the CA study site, whereas most non-Hispanic Whites were in IL. Our previous study with Latina perinatal women showed a significant and negative association between SRH and depressive symptoms and diabetes diagnosis in pregnancy. However, the mechanisms that explain those associations are not clear. Despite having higher depressive symptoms that were marginally significant (*p* = 0.055) and slightly lower SRH, it is also unclear why there was no significant association between depressive symptoms and SRH in the IL sample. While some research shows differences in SRH by race and ethnicity, with non-Hispanic Whites exhibiting higher scores, there may be contextual factors (e.g., rurality) that may have negatively affected the IL sample in our study. Therefore, future studies should account for contextual factors in and outside the healthcare setting.

As with standard practice for assessing glucose control in pregnancy, there are many benefits to evaluating the presence of depressive symptoms during this critical period. First, it is one of the few periods in a person’s life when they have numerous opportunities to detect elevated depressive symptoms in a relatively short time. Measuring depressive symptoms over time is critical to identify patients with worsening depressive symptoms. Our study offered a snapshot during the third trimester of pregnancy but also highlighted the role depressive symptoms can have in diabetes self-efficacy in the late stages of gestation. The results also show that SRH may vary by clinical population or setting. Our findings suggest that SRH may be an intervention point for some people, such as Latinas. However, this speculation should be tested with a larger, more diverse sample drawn from similar settings to account for potential healthcare characteristics.

Few studies have examined the associations between depressive symptoms, SRH, and diabetes self-efficacy in pregnant persons. One of the few studies found that pregnant persons with depressive symptoms or diabetes had worse SRH than their counterparts [17]. This is a critical population to study because SRH has been shown to decline in gestation [17]. Thus, it is crucial that clinicians assess patients’ SRH early in pregnancy and preferably before or in concert with diabetes testing to increase disease education to help identify potential intervention points.

### Limitations

This cross-sectional study presents several strengths but is also not without limitations. First, our study included a cross-sectional convenience sample of pregnant people with diabetes. The final sample was smaller than the target sample of 405 intended to detect significant mediated effects [47]. Nevertheless, this study yielded findings that merit further investigation, such as the potential role of clinical care settings and patient race and ethnicity. Therefore, future studies should replicate our design with a larger sample and equal proportions of racial and ethnic individuals from a similar clinical setting to test the role of race and ethnicity. Future studies should also consider examining the role of clinical care settings (e.g., context, treatment approaches) to determine the mechanism that might drive the moderating effect of the study site on the mediating role of SRH between depressive symptoms and diabetes self-efficacy. Second, we did not confirm a clinical diagnosis of depression using diagnostic measures with a clinical assessment. Additionally, depressive symptoms were measured once in late pregnancy. As mentioned, future studies should assess depressive symptoms early in pregnancy and preferably before diabetes testing, and additional assessments of a patient’s mental health can be conducted over time to identify potential escalation in symptoms. Clinicians and researchers can measure self-efficacy when patients are diagnosed with diabetes and throughout gestation to assess changes that can inform patient care. Third, we did not use directed acyclic graphs to identify the covariates for the model. Fourth, as described above, recruitment included patients with different types of diabetes. Related, we did not collect International Classification of Diseases (ICD) codes. Therefore, future studies should capture ICD codes and consider comparing equal proportions of birthing people by type of diabetes and test the associations reported.

## 5. Conclusions

The research shows that achieving normoglycemia in people with diabetes during pregnancy can reduce adverse pregnancy outcomes and improve the long-term health of both mother and child [48,49,50]. However, achieving and maintaining glycemic control requires significant commitment and behavioral changes on the part of the patient, which the presence of depression can influence. Here, we found that SRH mediated the association between depressive symptoms and diabetes self-efficacy in one clinical setting that consisted mainly of Hispanic/Latinas. While generalizability is limited, these findings suggest that further research is needed to understand the role of contextual (i.e., clinical setting) and individual-level factors (e.g., race and ethnicity) that moderate the mediating effect of SRH on the association between depressive symptoms and diabetes self-efficacy.

## Figures and Tables

**Table 1:** Sample demographic characteristics (*n* = 137).

Characteristics	CA Site (*n* = 82)	IL Site (*n* = 55)	Total (*n* = 137)
Age M (SD)	31.23 (6.58)	29.35 (5.57)	30.47 (6.24)
Age of diabetes diagnosis M (SD)	29.98 (6.79)	27.00 (7.77)	28.60 (7.38)
Language; *n* (%)			
English	51 (62.2)	55 (100)	106 (77.4)
Spanish	31 (37.8)	-	31 (22.6)
Race/Ethnicity; *n* (%)			
White, non-Hispanic	2 (2.4)	39 (70.9)	41 (29.9)
Black, non-Hispanic	5 (6.1)	9 (16.4)	14 (10.2)
Hispanic/Latina	69 (84.1)	3 (5.5)	72 (52.6)
Asian	4 (4.9)	2 (3.6)	6 (4.4)
Biracial	2 (2.4)	2 (3.6)	4 (2.9)
Marital Status; *n* (%)			
Married	36 (45.6)	24 (43.6%)	60 (44.8)
Cohabitating	18 (22.8)	13 (23.6)	31 (23.1)
Single/Separated	25 (31.6)	18 (32.7)	42 (31.3)
Employment; *n* (%)			
Full-time employed	19 (25.7)	26 (48.1)	45 (35.2)
Part-time employed	16 (21.6)	13 (23.6)	29 (22.7)
Not currently employed	39 (52.7)	15 (27.8)	54 (42.2)
Education; *n* (%)			
Less than high school	17 (21.5)	8 (14.5)	25 (18.2)
High school/GED or equivalent	27 (34.1)	17 (30.9)	44 (21.1)
Some College/Associate’s Degree	31 (39.2)	21 (38.2)	52 (38.0)
Bachelor’s degree or more	4 (5.0)	9 (16.4)	13 (9.5)
Income; *n* (%)			
No income	5 (6.3)	1 (1.8%)	6 (4.4)
Less than $15,000	14 (17.7)	12 (21.8)	26 (19.4)
$15,000 to $29,999	26 (32.9)	15 (27.3)	41 (30.5)
$30,000 to more	19 (24.0)	21 (38.2)	40 (29.8)
Declined to answer	11(13.9)	6 (10.9)	17 (12.6)
History of depression diagnosis; *n* (%)			
No	68 (85.0)	25 (45.5)	93 (68.9)
Yes	9 (11.3)	27 (49.1)	36 (26.7)
Unsure	3 (3.8)	3 (5.5)	6 (4.4)
EPDS cut-off; *n* (%)			
Not at risk for depression	68 (82.9)	44 (80.0)	112 (81.8)
At risk for depression	9 (11.0)	10 (18.2)	19 (13.9)

Note: All reports are based on available data for each categorical variable.

**Table 2:** Descriptive statistics for depressive symptoms, self-efficacy, and self-rated health.

	CA	IL	Total	Independent *t*-Test
	(*n* = 82)	(*n* = 55)	(*n* = 137)	
EPDS score; M (SD)	4.57 (4.32)	6.06 (4.32)	5.18 (4.37)	*t* (129) = −1.935, *p* = 0.055
DSES score; M (SD)	8.48 (1.58)	7.40 (1.69)	8.05 (1.70)	*t* (133) = 3.789, *p* < 0.001
SRH; M (SD)	3.00 (1.10)	2.80 (0.91)	2.92 (1.03)	*t* (134) = 1.117, *p* = 0.266

**Table 3:** Unstandardized estimates from the mediation analysis.

				95% CI (Bias Corrected)	
	Path	Estimate	S.E.	Lower 2.5%	Upper 2.5%
EPDS → SRH	a	−0.09	0.019	−0.129	−0.053
SRH → DSES	b	0.407	0.157	0.099	0.715
EPDS → DSES	c’	−0.075	0.035	−0.148	−0.009
Total indirect effect		−0.037	0.016	−0.077	−0.011

**Table 4:** Unstandardized estimates from the moderated mediation analysis.

				95% CI (Bias Corrected)	
	Path	Estimate	S.E.	Lower 2.5%	Upper 2.5%
EPDS → SRH	a	−0.236	0.059	−0.359	−0.126
SRH → DSES	b	0.409	0.157	0.101	0.716
EPDS → DSES	c’	−0.074	0.036	−0.148	−0.008
EPDS × Site → SRH	a1	0.103	0.039	0.028	0.182

## Data Availability

The data presented in this study are available upon request from the corresponding author.
